# Oral microbiota carriage in patients with multibracket appliance in relation to the quality of oral hygiene

**DOI:** 10.1186/s13005-016-0125-x

**Published:** 2016-10-28

**Authors:** Katharina Klaus, Johanna Eichenauer, Rhea Sprenger, Sabine Ruf

**Affiliations:** 1 Department of Orthodontics, Justus-Liebig University Giessen, Schlangenzahl 14, 35392 Giessen, Germany; 2 Private orthodontic practice, Rosengasse 2, 35305 Grünberg, Germany; 3 Private orthodontic practice, Marktgasse 2, 72070 Tübingen, Germany

**Keywords:** *Candida*, *Streptococcus mutans*, *Lactobacilli*, Oral hygiene, Fixed appliance, White spot lesions

## Abstract

**Background:**

The present study aimed to investigate the prevalence of oral microbiota (*Candida* species (spp.), *Streptococcus mutans*, and *Lactobacilli*) in patients with multibracket (MB) appliances in relation to the quality of oral hygiene.

Saliva and plaque samples were collected from three groups of 25 patients each (good oral hygiene (GOH), poor oral hygiene (POH), and poor oral hygiene with white spot lesions (POH/WSL)). Counts of colony forming units (CFU) of the investigated oral microbiota were compared using Chi-square and Mann–Whitney U tests.

**Results:**

Both saliva and plaque samples showed a high prevalence of *Candida* spp. in all patients (saliva: 73.4 %, plaque: 60.9 %). The main *Candida* species was *C. albicans*. The salivary CFU of *Candida* spp. in the GOH group was significantly lower than that in the POH group (*p* = 0.045) and POH/WSL group (*p* = 0.011). *S. mutans* was found in the saliva and plaque samples of all patients. *Lactobacilli* were found in the saliva samples of all patients and in 90.7 % of the plaque samples. In the saliva samples, the CFU of *Lactobacilli* were more numerous in the POH and POH/WSL groups than in the GOH group (*p* = 0.047).

**Conclusions:**

The investigated sample of patients showed a high carriage of oral *Candida* spp. Patients with WSL formation during MB appliance treatment exhibited higher counts of *Candida* and *Lactobacilli* compared with patients with good oral hygiene. Independent of oral hygiene quality, *S. mutans* was detected in all patients.

## Background

The insertion of a multibracket (MB) appliance induces a change in the number and composition of oral microflora. Besides the increase in potential cariogenic bacteria such as *Streptococcus mutans*, *Lactobacilli*, and periodontal pathogenic microorganisms, a marked increase in oral yeasts also occurs [1–6]. In particular, colonization with *Candida albicans* is of interest for orthodontists because of the fungus’ possible cariogenic effect [7–10], which has been demonstrated in vitro [11–13] and in vivo [14–20].

During orthodontic treatment with MB appliances, the development of white spot lesions (WSL) is an undesirable side effect with an incidence rate of 30 %–70 % [6, 21, 22]. WSL can develop as quickly as within 4 weeks [23]. Although patients with poor oral hygiene (POH) during treatment are reportedly affected [6, 24, 25], WSL are quite unpredictable from a clinical perspective. Some patients develop WSL despite acceptable oral hygiene, while others with consistently POH remain unaffected.

Therefore, the present pilot study aimed to analyze and compare oral microbiota in patients with MB appliances, with special emphasis on *Candida* spp. colonization. The quality of oral hygiene and the development of WSL were considered in this study. The null hypothesis was no difference in the amount of *Candida*, *S. mutans*, and *Lactobacilli* colonization among patients with good oral hygiene (GOH), POH, or POH with WSL.

## Methods

Ethical approval was granted by the ethical committee of the medical faculty of the Justus-Liebig-University of Giessen, Germany (No. 95/08).

Patients were selected from the Department of Orthodontics at Justus-Liebig-University Giessen, Germany. In general, after placement of a MB appliance, all patients of the department received the same standardized oral care instructions regarding frequency, technique, and duration of daily tooth brushing with fluoridated toothpaste, use of interdental brushes, and additional weekly fluoride gel application.

The inclusion criteria comprised patients aged 11 years or older without craniofacial anomalies, general diseases, or drug intake undergoing active MB appliance treatment in both jaws for at least three months. All selected patients were prospectively monitored for the quality of their oral hygiene at three consecutive regular appointments.

Dental plaque at selected index teeth (13, 21, 24, 33, 41, and 44) was visually inspected, and oral hygiene was categorized as follows:

1. GOH: no visible dental plaque on index teeth and no signs of gingival inflammation.
2. Average oral hygiene (AOH): small amounts of dental plaque on index teeth and mild signs of gingival inflammation.
3. POH: massive dental plaque on index teeth especially between the bracket and gingival margin, as well as marked signs of gingival inflammation.
4. POH with new WSL (POH/WSL): massive dental plaque on index teeth especially between the bracket and gingival margin, as well as marked signs of gingival inflammation and development of WSL during MB treatment. Possible pre-orthodontic WSL were excluded by analyzing the pre-treatment intraoral images.

Patients with consistent scorings of GOH or POH with/without WSL formation for three consecutive appointments were considered for possible inclusion in the present study. Patients with AOH were excluded. Written informed consent was obtained from the patients and their parents. For each group (GOH, POH, and POH/WSL), the first 25 patients fulfilling the abovementioned criteria were included.

The study appointment occurred during the next regular control appointment. Patients were instructed to stop oral hygiene for 24 h before the appointment (tooth brushing/antibacterial oral rinsing) to ensure a sufficient amount of plaque for sampling even in patients with GOH.

The dental status was checked using a slight modification of the DMF-T-index [26]. The index was modified to assess orthodontic patients. Tooth agenesis, extractions caused by orthodontic treatment plan, and impacted teeth were not scored as missing teeth.

The plaque samples were obtained by sterilized swabs (Nerbe plus, Winsen/Luhe, Germany) from the enamel surfaces along the gingival margin of the index teeth. In all patients of the POH/WSL group, a second plaque sample was obtained from the affected WSL surface regions with a probe tip and then applied on a second swab.

Saliva secretion was stimulated by chewing a paraffin wax pellet over a period of 5 min. The entire amount of saliva produced during that period was collected in a single-use plastic cup. From this sample, 2 ml of saliva was extracted using a sterile syringe (B. Braun, Melsungen, Germany) and used for further analysis.

*Candida* counts were identified with Sabouraud agar (Merck, Darmstadt, Germany) for the saliva samples. Depending on the number of colony forming units (CFU) per milliliter of saliva, counts were categorized into 0 (none), 1 (isolated, <10 CFU/ml), 2 (moderate, 10–10^2^ CFU/ml), 3 (many, 10^2^–10^3^ CFU/ml), and 4 (massive, >10^3^ CFU/ml). *Candida* species were differentiated by an Auxacolor™ 2-Test (Bio-Rad, Marnes-la-Coquette, France). Furthermore, the amounts of *S. mutans* and *Lactobacilli* were counted using the CRT® bacteria test (Ivoclar Vivadent, Schaan, Liechtenstein). The number of CFU per milliliter of saliva was categorized as 0 (none), 1 (moderate, <10^5^ CFU/ml), and 2 (many, ≥10^5^ CFU/ml). For plaque samples, the test manufacturers recommended classifying the occurrence of *S. mutans*, *Lactobacilli*, and *Candida* in terms of a binary (yes/no) decision only, because the plaque samples have no defined volume.

Statistical analysis was performed by IBM SPSS Statistics 22 (IBM Company, Chicago, IL, USA). Descriptive data analysis was conducted by exploring means, standard deviations, minima, and maxima. The data were not normally distributed, and the differences between groups were assessed using the Chi-square and Mann–Whitney U tests.

## Results

The general descriptive characteristics of the final patient sample are given in Table 1. The mean age of the total sample was 14.4 ± 1.5 years and did not differ significantly among the three oral hygiene groups. The gender distribution was nearly equal in the total sample and in the POH/WSL group. Female predominance was found in the GOH group, whereas male predominance was noted in the POH group. The DMF-T-index was generally very low (mean range: 0–0.1) and did not differ among the oral hygiene groups.

**Table 1:** Demographic characteristics of the studied patient sample (*n* = 75) and their fixed appliance treatment duration at the time of the study

Group	Age (years) mean ± SD	Male	Female	MB in situ (months) mean ± SD
Total sample	14.4 ± 1.8	36	39	16 ± 9.2
GOH	14.6 ± 1.8	6	19	14.9 ± 7.9
POH	14.0 ± 1.4	16	9	13.4 ± 6.7
POH/WSL	14.6 ± 1.3	14	11	19.6 ± 11.3

*SD* standard deviation, *MB* multibracket appliance, *GOH* good oral hygiene, *POH* poor oral hygiene, *POH/WSL* poor oral hygiene with white spot lesions

At the time of saliva and plaque sample collection, the mean wearing time of the MB appliance in the GOH group (14.9 ± 7.9 months) was insignificantly higher than that in the POH group (13.4 ± 6.7 months). The POH/WSL group showed the longest wearing period (19.6 ± 11.3 months) with a significant difference from the other groups (*p* = 0.017).

**Fig. 1:**
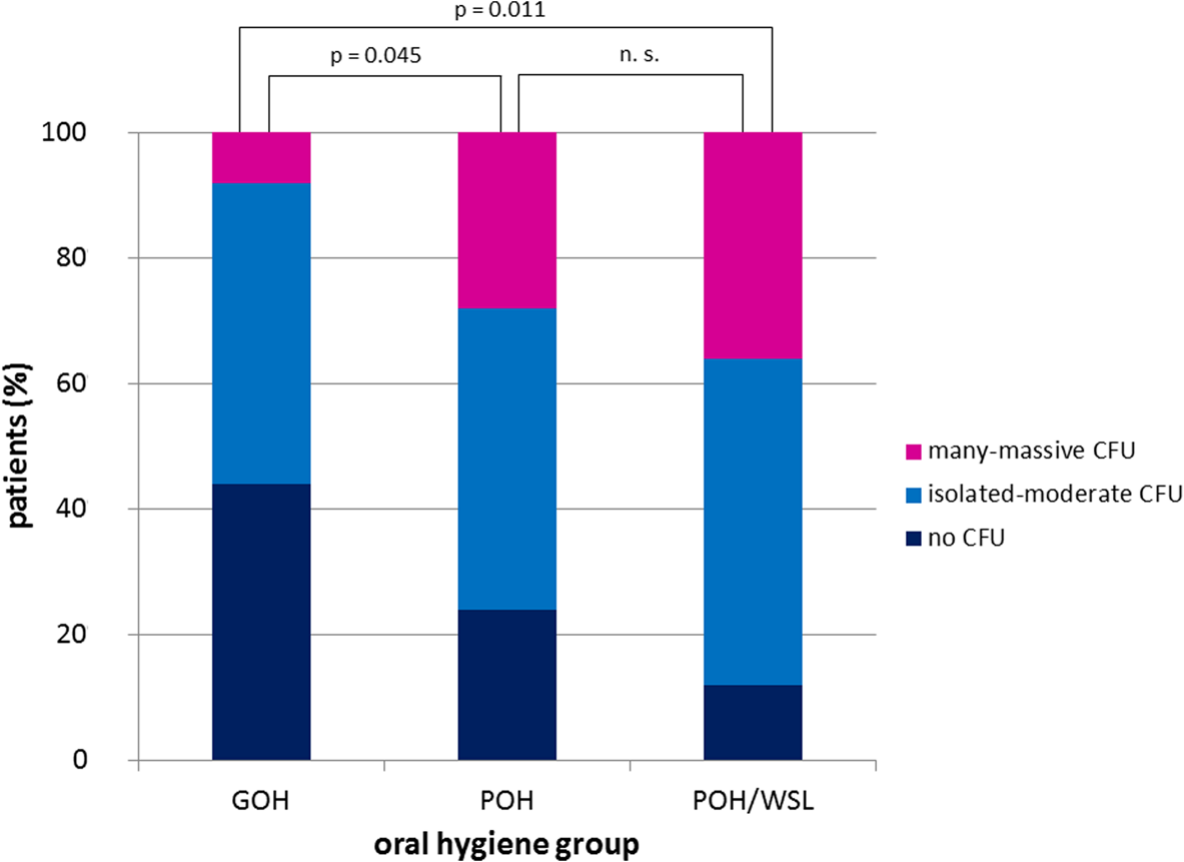
Relative *Candida* carriage in patients’ saliva in relation to the oral hygiene groups. A significant difference was found between the GOH and POH groups (*p* = 0.045), as well as between the GOH and POH/WSL groups (*p* = 0.011)

Analysis of the saliva samples revealed that 73.4 % of the patient sample were *Candida* carriers. About half of the carrier samples (49.4 %, *n* = 37) showed isolated to moderate numbers of CFU, whereas 24 % (*n* = 18) presented many to massive CFU. CFU of *Candida* spp. were significantly lower in the GOH group than in the POH group (*p* = 0.045) and POH/WSL group (*p* = 0.011) (Fig. 1). Saliva samples of *Candida* carriers (*n* = 54) showed *C. albicans* in 83.3 % (*n* = 45), *C. dubliniensis* in 14.8 % (*n* = 8), and *C. albicans II* in 1.9 % (*n* = 1). No significant difference in the distribution of species was found among the different oral hygiene groups.

**Fig. 2:**
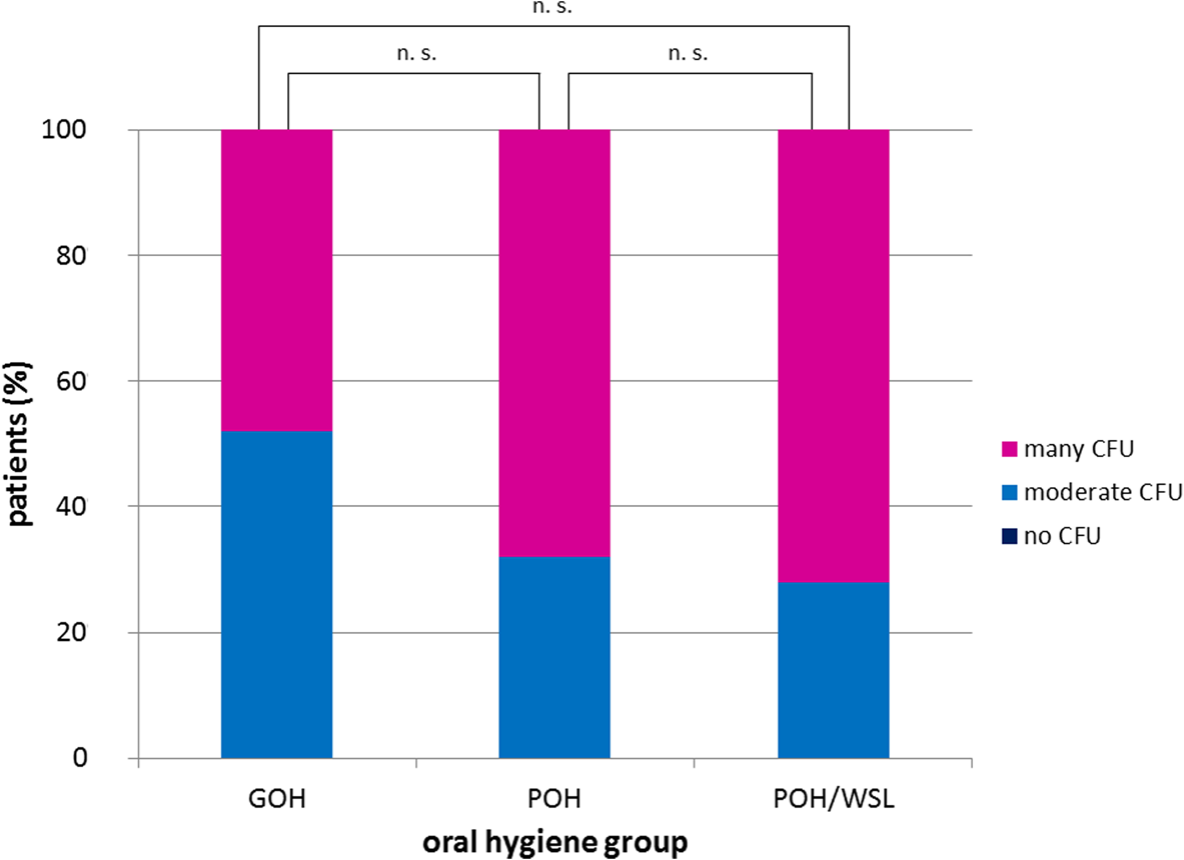
Relative *S. mutans* carriage in patients’ saliva in relation to the oral hygiene groups. No significant difference was found among the groups

**Fig. 3:**
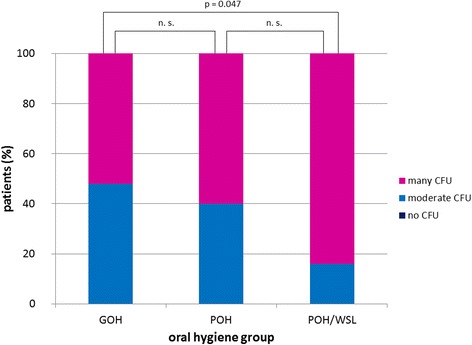
Relative *Lactobacilli* carriage in patients’ saliva in relation to the oral hygiene groups. A significant difference was observed between the GOH and POH/WSL groups (*p* = 0.047)

*S. mutans* and *Lactobacilli* were detected in the saliva samples of all patients. Patients with POH irrespective of WSL formation presented higher counts of *S. mutans*, but the difference was not statistically significant (Fig. 2). High *Lactobacilli* CFU were noted in 52 % of patients in the GOH group. A comparable amount of CFU was found in 60 % and 84 % of patients in the POH group and POH/WSL group, respectively. This difference was statistically significant (*p* = 0.047; Fig. 3). For the saliva samples, the null hypothesis regarding *S. mutans* was accepted but rejected in *Candida* and *Lactobacilli*.

Analysis of the plaque samples revealed a 60.9 % prevalence of *Candida* carriers in the total sample. In the POH/WSL patients, the plaque samples from the index teeth showed an insignificantly higher *Candida* prevalence (62.4 %). The second plaque samples from the affected white spot enamel regions exhibited only a slightly and insignificantly different prevalence of 64 %.

Among the *Candida* carriers (*n* = 46), 82.6 % (*n* = 38) of the carriers showed *C. albicans*, 15.2 % (*n* = 7) presented *C. dubliniensis*, and *C. albicans II* was found in 2.2 % (*n* = 1). As in the saliva samples, the distribution of *Candida* species revealed no statistically significant difference among the oral hygiene groups. Similar to the saliva samples, *S. mutans* was verified in the plaque samples of all patients, whereas *Lactobacilli* were found in 90.7 % (*n* = 68) of all plaque samples. For the plaque samples, the null hypothesis regarding all types of microbiota was accepted.

## Discussion

Counts of oral microbiota peak at about three months after insertion of a fixed appliance [3, 27–30]. Although most studies [3, 28–30] reported a slight decrease thereafter, Arslan et al. [27] found a consistent increase in *Candida*, *S. mutans*, and *Lactobacilli* counts up to 12 months of treatment. Despite the slight decrease reported by the other studies, patients showed consistently higher counts of oral microbiota compared with pre-treatment [28–30]. In the present patient sample, the mean treatment duration at the study appointment ranged between 13.4 and 19.6 months with a significantly longer treatment time in the POH/WSL group, which was far longer than that in all other studies in the literature. For the POH/WSL group in the present study, the influence of oral microbiota or longer treatment duration on the development of WSL could not be clarified. However, given the cross-sectional design of the present study, the development of microbial counts in all the investigated groups during the treatment period remains unclear.

In the current literature, oral *Candida* carrier rates vary between 28.6 % and 57.2 %, depending on the age of the examined individuals and the microbiological sampling method [31–36]. During orthodontic treatment, an even greater variation with prevalence rates between 8.3 % and 78.8 % was reported [1, 3, 27, 37]. The results of the present study corresponded to the top end of these data with *Candida* prevalence in dental plaque of 60.9 % and in saliva of 73.4 % of the patients.

Unfortunately, no standard currently exists for oral microbiological sampling. According to the literature, the most popular sampling methods are stimulated and unstimulated saliva samples, centrifuged saliva samples, swabs from oral mucosa, plaque samples, and imprint cultures from different sites in the oral cavity [20]. When comparing results achieved by different sampling methods and different culture media [38], centrifuged saliva samples and imprint cultures showed a significantly higher sensitivity in detecting oral yeasts [39] than cultures from the other sampling methods. In the current literature, studies with patient samples comparable with the present investigation [1, 3, 27, 37] used various sampling methods (Table 2). Hägg et al. [3] used three different sampling methods, and the method closest to the sampling collection in our study (pooled plaque) revealed a clearly lower *Candida* rate (22.2 %), in contrast to our study. Furthermore, 18.5 % of their patients became *Candida* carriers during treatment. In the present work, the incidence rates of *Candida* infection could not be revealed because of the cross-sectional design of the study.

**Table 2:** Oral microbiological sampling methods and investigated time points used in previous studies to analyze *Candida* carriage in orthodontic patients

Study group	Time points of investigation		Type of appliance		Saliva sampling	Plaque sampling	Oral mucosa sampling
	Before insertion	During treatment	Fixed	Removable			
Current study	-	**x**	**x**	-	**stimulated saliva**	**pooled plaque**	-
Addy et al. 1982 [1]	-	**x**	**x**	x	-	-	imprint culture
Hägg et al. 2004 [3]	x	**x**	**x**	-	oral rinse	**pooled plaque**	imprint culture
Arslan et al. 2008 [27]	x	**x**	**x**	-	**stimulated saliva**	**pooled plaque**	-
Arendorf, Addy 1985 [37]	x	**x**	-	x	-	-	imprint culture

Comparable study methods are highlighted in bold text

In concordance with the present findings, the current literature on oral yeast carriage indicated *C. albicans* as the dominant species both in orthodontic-treated patients [3, 27, 37] and untreated individuals [31, 33]. In our sample, *C. dubliniensis* was the second most frequent species and one patient presented *C. albicans II*; the literature reported other additional *Candida* strains. Given that *C. albicans* and *C. dubliniensis* present pronounced morphological similarities and some common tests are unable to differentiate them reliably [16], *C. dubliniensis* was possibly undetected in other investigations.

In general, the current literature agreed that *S. mutans* and *Lactobacilli* are characteristic for deep carious lesions [14, 15, 40–42] and therefore not for initial carious lesions such as WSL. Aas et al. [40] verified high concentrations of *Actinomyces* and non-*S. mutans–Streptococci* in initial lesions, but they additionally emphasized that bacterial profiles in initial lesions are more complex than in advanced destruction stages. Badet and Thebaud [41] revealed a strong correlation between the DMF-T-index and amount of *Lactobacilli* counts and furthermore showed that *Lactobacilli* are not isolated from initial carious lesions because of weak adherence potential; retentive niches for adherence are required by *Lactobacilli*. A fixed orthodontic appliance inevitably presents retentive niches, so whether the high *Lactobacilli* counts in the present study are associated with WSL development or simply caused by better possibilities for plaque accumulation remains unclear.

However, an in vivo study by Arneberg et al. [43] revealed higher bacterial counts for *S. mutans* and *Lactobacilli* at initial carious lesion surfaces compared with the unchanged control surfaces of neighboring teeth, suggesting the participation of these microorganisms even in initial enamel lesions.

The association of *C. albicans* with dental caries has been frequently investigated in patients with severe early childhood caries [17–19, 44–46]. Current in vitro and in vivo studies [12, 13] demonstrated a symbiosis between *C. albicans* and *S. mutans* in dental plaque, in which the two microbiota strains stick together because of extracellular polysaccharides. During the maturation of dental plaque, large microcolonies of *S. mutans* and *C. albicans* can be observed after 42 h [13]. Falsetta et al. [13] investigated the effect of *S. mutans* and *C. albicans* in rodents and showed that co-infected animals present significantly more severe carious lesions, whereas rodents infected with either *S. mutans* or *C. albicans* revealed initial carious lesions. In the present study, a statistically significant relationship between the quality of oral hygiene and *Candida* colonization could only be demonstrated for the saliva samples. Furthermore, the present patient sample showed high counts of *S. mutans* and *Lactobacilli* in POH or POH/WSL patients (significant for *Lactobacilli* in saliva only). In other words, all three microorganisms investigated in the present study influenced caries development, but the relative amount of their contribution and possible timing of their contribution remain unknown.

The first possible limiting factor of the present study is the lack of precise knowledge on the age and maturity of the dental plaque analyzed, because one cannot be sure that all patients followed the instructions to stop their oral hygiene for 24 h before the study appointment. Even if the patients followed this instruction, we are uncertain of the events that occurred during the previous days. Additionally, patients received no standardized information regarding eating or drinking between the appointments and before sample collection. Perhaps the comparability could have been improved slightly if all patients had a professional cleaning of their teeth, followed by the sampling appointment after a determined time period.

Furthermore, we cannot exclude the Hawthorne effect, because the knowledge about study participation and the special reminders about the study appointment given by the study team can evoke patient’s behavior modification [47]. Moreover, such knowledge and reminders possibly influenced oral hygiene and in turn the oral microbiota in the weeks before study sampling.

One could also question the categorization of the patients into the GOH and POH groups based on the subjective judgement of the treating orthodontists instead of an objective measurement. However, even with objective measurements, cut-off values for the groups have to be defined, which still provide ample space for large variations in the quality of oral hygiene. Nevertheless, a quantitative plaque assessment would have improved the comparability with the results of Hägg et al. [3] and Arslan et al. [27].

Given the present results, oral *Candida* counts and *S. mutans* or *Lactobacilli* counts showed no clear interrelation to the development of WSL. The current literature revealed that the abovementioned organisms participated in initial enamel caries formation. Further prospective longitudinal clinical trials concerning changes in oral microbiota during MB treatment are required to clarify the developmental process of WSL.

## Conclusions

The investigated sample of patients showed a high carriage of oral *Candida* spp., *S. mutans*, and *Lactobacilli*. Patients with WSL formation during MB appliance treatment showed higher counts of *Candida* and *Lactobacilli* compared with patients with GOH. Independent of oral hygiene quality, *S. mutans* was detected in all patients.

## Abbreviations

AOH: Average oral hygiene
CFU: Colony forming units
GOH: Good oral hygiene
MB: Multibracket
POH: Poor oral hygiene
POH/WSL: Poor oral hygiene with white spot lesions
Spp: Species
WSL: White spot lesions
